# Effects of intravenous d9-THC on pupillary reaction and pupil size: a prospective, placebo-controlled trial in healthy volunteers not regularly consuming cannabis

**DOI:** 10.1186/s12886-025-04107-7

**Published:** 2025-05-13

**Authors:** Maren Kleine-Brueggeney, Fritz Priemer, Frank Konietschke, Lorenz Theiler, Robert Greif

**Affiliations:** 1 Department of Cardiac Anesthesiology and Intensive Care Medicine, Deutsches Herzzentrum der Charité (DHZC), Augustenburger Platz 1, 13353 Berlin, Germany; 2 Charité - Universitätsmedizin Berlin, corporate member of Freie Universität Berlin and Humboldt- Universität zu Berlin, Berlin, Germany; 3 Office of Medical Experts, Wonneberg, Germany; 4 Institute of Biometry and Clinical Epidemiology, Charité - Universitätsmedizin Berlin, corporate member of Freie Universität Berlin, Humboldt-Universität zu Berlin, Berlin, Germany; 5 Department of Anaesthesiology, Cantonal Hospital Aarau, Aarau, Switzerland; 6 Department of Surgical Science, University of Torino, Torino, Italy

**Keywords:** Tetrahydrocannabinol, Pupillography, Pupillary reaction, Pupils, Legal medicine

## Abstract

**Introduction:**

This prospective, single-blind, placebo-controlled trial examined the effects of intravenous delta-9-tetrahydrocannabinol (d9-THC) on the pupillary light reflex and pupil size in volunteers not regularly consuming cannabis.

**Methods:**

With ethics committee approval and written informed consent, healthy cannabis-naïve or abstinent volunteers were included. Fifteen volunteers received an intravenous bolus of d9-THC and four received placebo. Pupillary reaction and pupil size were assessed by pupillography before and for 5 h after drug administration. Primary outcome was relative amplitude. Secondary outcomes were latency, velocity of contraction, constriction time, contraction amplitude, and pupil diameter.

**Results:**

Pupillographic measurements were significantly altered by THC: The relative amplitude was significantly reduced with a global difference between groups (*p* = 0.001). The relative amplitude significantly declined at 20 min after THC administration (23.5–15.0%), and stayed constant in after placebo (27.5–28.1%). Constriction time was significantly reduced with a significant global time effect (*p* = 0.002), global group effect (*p* = 0.001), and global effect of the interaction between group and time (*p* < 0.001). Contraction amplitude was reduced with a significant global group effect (*p* < 0.001). Latency and velocity of contraction demonstrated a statistically non-significant increase. Pupil size decreased after THC administration.

**Discussion:**

Pupillography can objectively detect effects of THC on the human eye. In cannabis-naïve or abstinent volunteers THC dampens the pupillary light reflex which could result in an increased sensitivity to light. THC does not cause mydriasis, but rather miosis. These results can substantiate questions regarding liability and driving ability under the influence of THC.

**Trial registration:**

The study was prospectively registered at www.isrctn.com (registration number ISRCTN53019164) on 14/04/2010.

## Introduction

Consumption of cannabis is increasing for both recreational and medical use. As a consequence, the physical and mental effects of delta-9-tetrahydrocannabinol (d9-THC or THC), the definition of “acting under the influence”, and the impact of THC consumption on liability and driving ability are a matter of debate. Many countries or states have established limits of THC serum concentrations or are debating such limits. Such discussions are substantiated by statistics of road traffic accidents [1–3], investigations regarding driving performance under the influence of THC [4, 5], and studies on the clinical effects of THC.

In terms of clinical effects, neurocognitive performance under the influence of the same amount of THC has been reported as significantly worse in occasional compared to heavy or regular cannabis users [6, 7]. Hence, the effects of THC likely not only depend on the mode and quantity, but also on the frequency of consumption. It might therefore be sensible to institute a more complex approach to the assessment of THC consumption that relies not only on serum concentrations, but also on an objective assessment of physical and mental effects and actual impairment from THC consumption. Such assessment of impairment could include both mental and somatic effects of THC, including the effects of THC on the eyes.

The assessment of pupil function has been described as a sensitive and useful indicator for being under the influence of substances acting on the central nervous system [8–10], and infrared pupillography has been described as a useful tool for police traffic checks [11]. According to police training programs, wide pupils that are sluggish in reaction to light are an indicator of consumption of THC. While it is often considered that pupils dilate in reaction to THC, a review on the clinical effects of THC on the eye reported inconsistent effects on pupil diameter: [12] Three studies reported an increase and five studies reported a decrease in pupil diameter, while another study did not find any significant variation in pupil diameter after THC consumption [12]. Furthermore, cannabis use has been reported to impact contrast sensitivity, particularly in cannabis users starting at an early age [13]. 

Because of the inconsistent results of published studies and the ongoing debate about the effects of THC on the human eye, we conducted this prospective, single-blind, placebo-controlled clinical trial to investigate the static and dynamic pupillary reaction to THC. This study was carried out in healthy volunteers not regularly consuming cannabis. After a single defined bolus of IV THC pupil diameter and the various parameters of the pupillary light reflex (PLR) were examined over time using a videopupillography system. The aim was to provide scientific and objective data about the effects of THC on pupil size and pupillary reaction to light in volunteers not regularly consuming cannabis.

## Materials and methods

### Study design and participants

This prospective, single-blind, placebo-controlled clinical trial used pupillography to assess the effects of THC and its metabolites on pupillary function and pupil size in healthy volunteers not regularly consuming cannabis. It was performed with ethics committee approval (Cantonal Ethics Committee Bern, approval number KEK 241–09) and approval of the relevant authorities/ bodies (Federal Office of Public Health of the Swiss Confederation, Swissmedic) at Bern University Hospital in Switzerland. All volunteers gave written informed consent and the study was registered at www.isrctn.com (registration number ISRCTN 53019164).

Participants were older than 18 years and were either cannabis naïve or cannabis abstinent for at least one month. Exclusion criteria were tobacco smoking within the last three months, suspected ischemic heart disease, cardiac arrhythmias, use of illicit drugs (e.g. heroin, cocaine, LSD), treated or suspected psychiatric disease at any point during their lifetime, pregnancy (test mandatory in females), any medication altering cytochrome P activity, and body mass index < 16 or > 35 kg m^− 2^. All volunteers underwent a standard urine drug screening procedure that would have detected cannabinoids, opioids, cocaine, barbiturates, benzodiazepines, and stimulants such as amphetamines before inclusion into the study.

This assessment of the effects of THC on the eye was an add-on study as part of a larger study on the pharmacokinetics and effects of IV THC in healthy volunteers. To allow for pharmacokinetic modelling of intravenous THC 306 healthy volunteers were screened for genetic variants of CYP2C9 and all volunteers expressing rare genetic variants of CYP2C9 as well as 5 wild-type carriers received THC. The study was therefore prospective, but not randomized. The pharmacokinetic results have already been published and demonstrated minimal effects of the genetic variants on THC pharmakokinetics [14]. 

### Study set up

The study took place in a fully equipped recovery room of Bern University Hospital which, on the days when the study was conducted, was solely used for the study to provide a stable environment with as little external stimuli as possible. All volunteers had an IV line and an arterial line placed in the radial artery of the non-dominant hand, placed under local anesthesia. The arterial line was used for real-time monitoring of blood pressure and blood sampling. All volunteers were continuously monitored (heart rate, ECG, SpO_2_, invasive blood pressure monitoring) using the medical equipment of the recovery room. Dedicated study personnel, including an anesthesiologist, was continuously present in the recovery room.

With full monitoring in place, volunteers received a single bolus of THC 0.1 mg/kg body weight intravenously or an equivalent volume of NaCl 0.9% as placebo, manually administered by a member of the study team using a syringe. THCPharma (Frankfurt am Main, Germany) provided the THC, which was prepared to its IV THC injection solution by the hospital pharmacy of the Inselspital, Bern, Switzerland according to the method of Naef et al. following good manufacturing practice [15]. The solution had a concentration of 1 mg/ml and was provided in vials of 10 ml. Volunteers were monitored for at least 5 h following IV injection of THC or placebo and were followed-up in person in the recovery room on day 1 and day 2 after the injection.

### Measurements

Demographic data of all volunteers were recorded, including sex, age, weight and height. Heparinized blood samples for the analysis of THC and its metabolites Hydroxy-THC (THC-OH) and Carboxy-THC (THC-COOH) were drawn from the arterial line before IV injection and at 1, 2, 5, 10, 15, 20, 30, 45, 90, 180, 300 min after the IV bolus. Plasma levels of THC, THC-OH and THC-COOH over time were used to establish a dedicated pharmacokinetic model of IV THC [14]. Pupillographic assessements were performed between 23 Jun 2010 and 05 Feb 2011.

### Pupillography

Pupillary measurements were taken at baseline (i.e. before IV injection of THC or placebo) and at 20, 60, 120, 180, 240, and 300 min after IV injection of THC or placebo.

Pupillographic testing was performed with the F^2^D infrared pupillograph from AMTech^®^ (AMTech Pupilknowlogy GmbH, Dossenheim, Germany) according to its instruction manual. The F^2^D measures the diameter of a pupil and the pupillary light reflex with an infrared line scan camera [11]. The wavelength of the emitted light was 880 nm, the measuring frequency 25 Hz and the resolution 0.05 mm. Completely darkened glasses within the integrated measuring apparatus covered both eyes of the volunteers and volunteers were advised to hold the eyes open without winking or any movement or fixation of the eyes. Time was given for the eyes to accommodate to the darkened glasses before the start of the pupillographic measurements.The duration of the light stimulus was 0.2 s. A luminance 255 (56 lx) was used as intensity of the light stimulus [16]. To ensure reproducible results and to minimize the effect of artifacts within the data due to winking or insufficient fixation of the eye with accommodation miosis, each measurement was performed until three unimpaired pupillometric curves were obtained. The average curve and its parameters were automatically calculated by the software “LoOK!”, integrated into the F^2^D system. Between each measurement, an interval of at least ten seconds was allowed to ensure adequate recovery of the pupils. Recording of the pupil diameter started the moment the stimulus of light was emitted. All measurements were done for the left pupil.

**Fig. 1:**
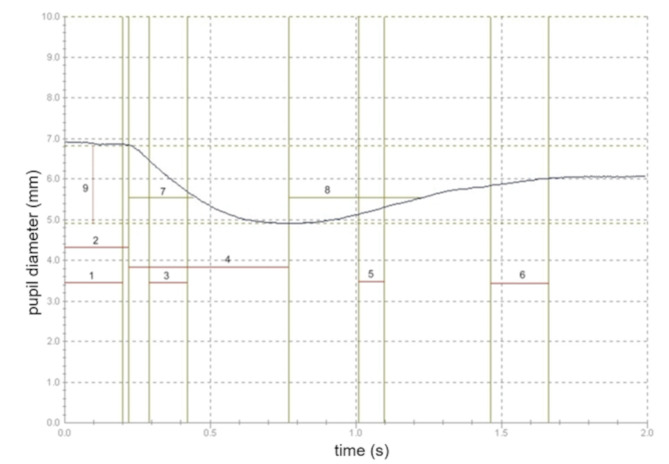
A typical pupillography curve. The following parameters are reported in this study: Initial diameter of the pupil (y-axis at time 0); Line 1: Length of stimulus (set to 200ms); Line 2: latency; Interval 3: velocity of contraction; Interval 4: constriction time; Line 9: contraction amplitude

A typical pupillography curve is shown in Fig. 1. The following functionally relevant parameters were assessed:

- Initial diameter: This is the diameter of the pupil before the stimulus and during latency.
- Latency (L): This is the time between the onset of the light stimulus and the beginning of the pupillary response. Normal latency is at least 200ms.
- Velocity of contraction (VC): The velocity of pupil contraction to a light stimulus is calculated from the mainly linear downward slope of the curve (interval 40–80%).
- Constriction time (T_C_): This is the time from onset of the pupillary constriction to minimal diameter.
- Contraction Amplitude: This is the difference between the initial diameter and the minimal diameter of the pupil.
- Relative (pupil constriction) amplitude: This is calculated as contraction amplitude/ initial diameter = (initial diameter - minimal pupil diameter)/ initial pupil diameter. It is given in percent.

For clarity, the relative (pupil constriction) amplitude is calculated as the pupil diameter in percent from baseline for any given timepoint, i.e. for the timepoint 20 min this will be the pupil diameter after the stimulus at 20 min in percentage of the pupil diameter directly before the stimulus at 20 min. This is done to control for interindividual differences in pupil diameter and to normalize for intraindividual fluctuations in pupil diameter. As such, the relative amplitude is a measure of the contractility of the pupil in response to light.

### Definition of outcome parameters and aim

***Primary outcome parameter*** was the relative amplitude of the pupillary light reflex.

***Secondary outcome parameters*** were the pupil diameter (i.e. the initial diameter before the light stimulus at each timepoint), and other parameters of the pupillary light reflex such as the latency, the velocity of contraction, the constriction time and the contraction amplitude.

#### Aim

The aim was to estimate the impact of IV THC on the function and size of the pupil.

### Statistical methods

#### Sample size

The main pharmacokinetic study was composed of a total of 25 volunteers receiving THC and 4 volunteers receiving placebo [14]. The present study on pupillography was started as an add-on to the main pharmacokinetic study with ethics committee approval of an amendment to the study protocol when the main pharmacokinetic study had already started. Hence, for practical reasons, a sample of 15 volunteers receiving THC and 4 volunteers receiving placebo were enrolled in the pupillography study. This was an exploratory study with no formal hypothesis testing or sample size calculation.

### Statistical analysis

We modeled the data with a factorial longitudinal design with whole-plot factor Treatment (placebo, THC) and Time as a sub-plot factor. Descriptive summaries of the outcomes per treatment group and time point are reported. We tested for global differences between the groups in terms of main treatment effect, main time effect and interaction effect using purely nonparametric rank-based methods for factorial longitudinal data. Marginal differences between time points or groups were assessed at a 5% level with rank-methods for testing equality of paired distributions or Wilcoxon-Mann-Whitney tests. We used the Bonferroni correction to adjust for multiplicity. Since the sample size of the placebo group (*n* = 4) is small, we focus on descriptive summaries and report results from inference methods purely exploratory. All data evaluations were conducted using R (version 4.2.2) using the nparLD and ggplot2 packages and core functions.

## Results

Demographic data are displayed in Table 1. Nine females and 6 males received THC, and 2 females and 2 males received placebo. All volunteers were under 30 years old. There were no statistically significant differences in the demographic data between the THC and the placebo group. Pupillographic data of one volunteer were excluded as measurements were accidentally performed using a luminence of 30 and 100, but not 255. Any other missing pupillographic data resulted from volunteers not being able to tolerate the light stimuli as they found them to be disturbingly bright. In 4 volunteers several consecutive measurements could not be performed due to an intolerance to the light stimulus, others did not tolerate the measurements at a single timepoint.

**Table 1:** Demographic data. Data are number (percent) or median (25%, 75% percentile))

	THC *n* = 15	Placebo *n* = 4	*p*-value
Sex: female/ male, n (%)	9/ 6 (60/ 40)	2/2 (50/50)	1.00
Age (years)	23.0 (21.0, 24.5)	22.5 (22.0, 23.5)	0.95
Height (cm)	172 (168,182)	180 (172, 183)	0.81
Weight (kg)	66.0 (61.0, 81.5)	77.0 (71.0, 83.8)	0.25
BMI (kg m^− 2^)	23.0 (20.9, 25.9)	25.0 (22.7, 27.6)	0.31

### Primary outcome: relative amplitude

**Fig. 2:**
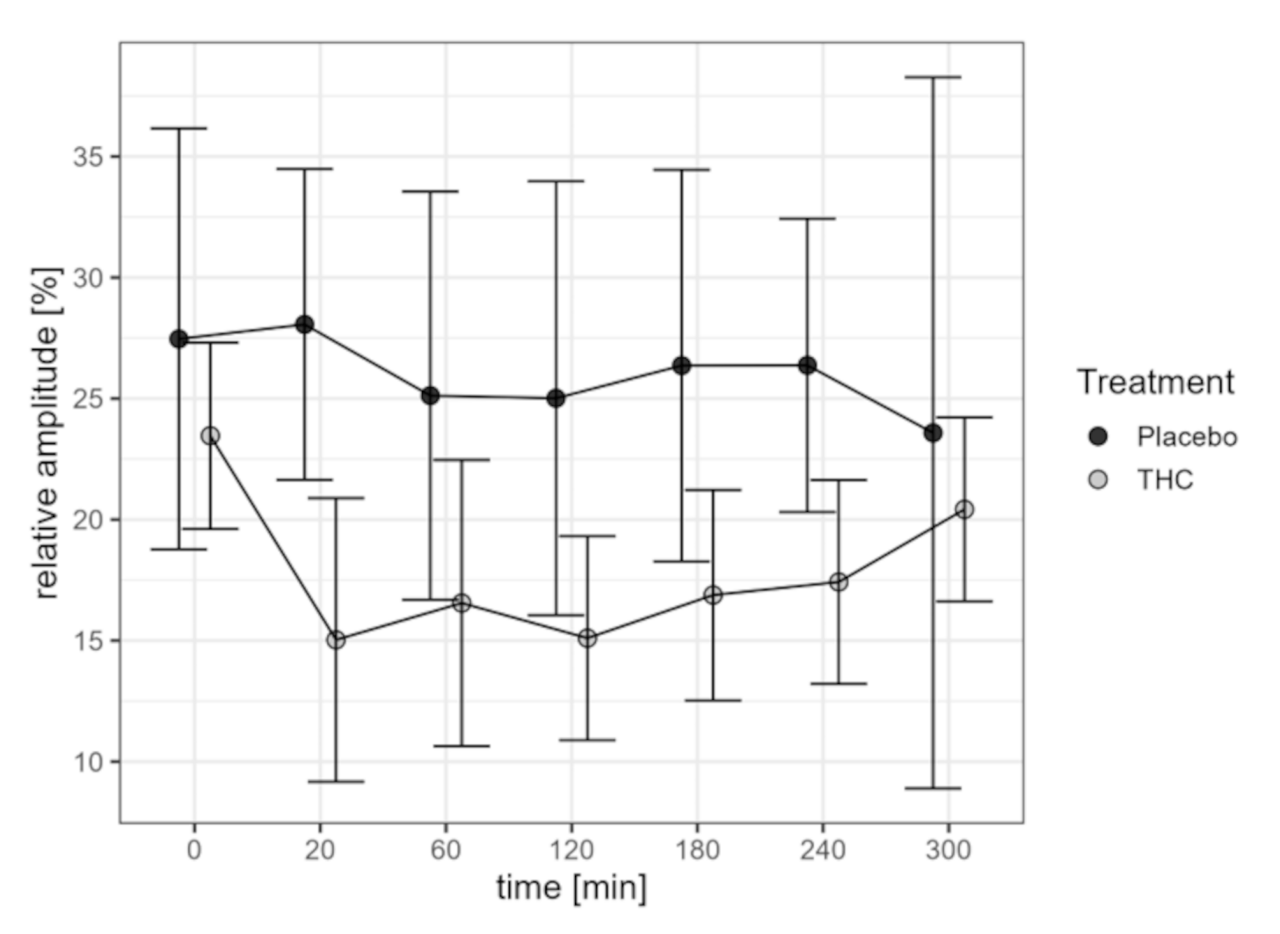
Relative amplitude in the placebo and THC group over time. Data reported are mean and 95% confidence intervals. Placebo: *n* = 4 for all time points; THC: baseline *n* = 14, 20 min *n* = 10, 60 min *n* = 7, 120 min *n* = 10, 180 min *n* = 13, 240 min *n* = 12, 300 min *n* = 13

The results of the measurements of the relative amplitude of the pupillary light reflex are given in Table 2 and are visualized in Fig. 2. The relative amplitude is lower in the THC group than in the placebo group at all time points. Also, values within the THC group are significantly lower at all time points after THC injection compared to baseline (*p* < 0.01 at 300 min and *p* < 0.01 for all other time points). Whereas the relative amplitude stays almost constant over time under placebo, it drops from 23.5 to 15.0% between baseline and 20 min after THC administration, then stays about constant until 120 min and slightly increases therefrom until 300 min, returning close to baseline.

The statistical tests reveal a global difference between the treatment groups (*p* < 0.01), whereas the data do not provide evidence of a time effect (*p* = 0.13) or an interaction effect of the interaction between time and group (*p* = 0.18). We note, however, that these non-significant results follow from the very small sample size in the placebo group.

**Table 2:** Relative amplitude in %. Reported data are mean with 95% ± standard deviation (confidence interval)

	Baseline	20 min	60 min	120 min	180 min	240 min	300 min
THC*	23.5 ± 6.7 (19.6; 27.3)	15.0 ± 8.2 (9.2;20.9)	16.6 ± 6.4 (10.6; 22.5)	15.1 ± 5.9 (10.9; 19.3)	16.9 ± 7.2 (12.5; 21.2)	17.4 ± 6.6 (13.2; 21.6)	20.4 ± 6.3 (16.6; 24.2)
Placebo*	27.5 ± 5.5 (18.8; 36.2)	28.1 ± 4.0 (21.6; 34.5)	25.1 ± 5.3 (16.7; 33.6)	25.0 ± 5.6 (16.1; 34.0)	26.4 ± 5.1 (18.3; 34.5)	26.4 ± 3.8 (20.3; 32.4)	23.6 ± 9.2 (8.9; 38.3)

* Placebo: *n* = 4 for all time points; THC: baseline *n* = 14, 20 min *n* = 10, 60 min *n* = 7, 120 min *n* = 10, 180 min *n* = 13, 240 min *n* = 12, 300 min *n* = 13

### Secondary outcome parameters

The detailed measurements of all secondary outcome parameters are given in Table 3.

### Pupil diameter

**Fig. 3:**
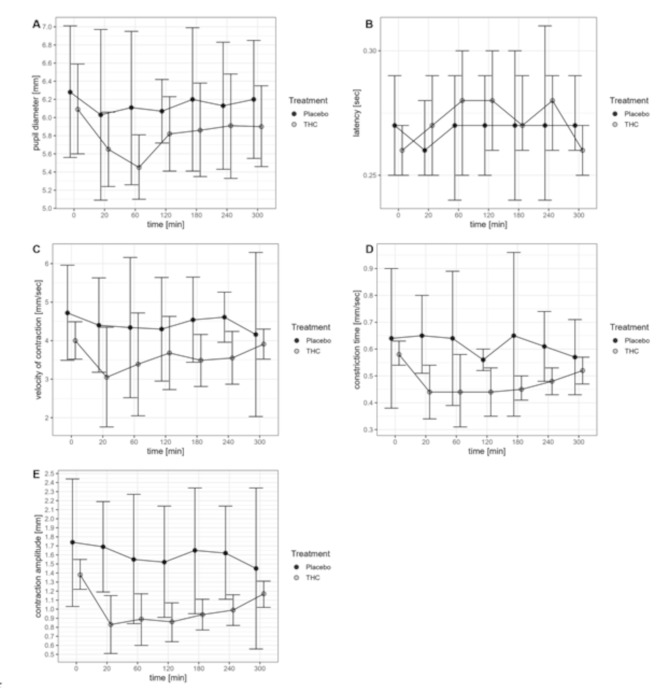
Pupil diameter (**A**), latency (**B**), velocity of contraction (**C**), constriction time (**D**) and contraction amplitude (**E**) over time for the THC and the placebo group. Data reported are mean and 95% confidence intervals. Placebo group: *n* = 4 for all timepoints and all parameters; THC group for pupil diameter (**A**), latency (**B**) and constriction time (**D**): baseline *n* = 14, 20 min *n* = 10, 60 min *n* = 7, 120 min *n* = 10, 180 min *n* = 13, 240 min *n* = 12, 300 min *n* = 13; THC group for velocity of contraction (**C**) and contraction amplitude (**E**): baseline *n* = 14, 20 min *n* = 8, 60 min *n* = 7, 120 min *n* = 10, 180 min *n* = 13, 240 min *n* = 12, 300 min *n* = 13

The diameter of the pupil before THC administration was 6.1 ± 0.9 mm in the THC and 6.3 ± 0.5 mm in the placebo group. The course of the pupil diameters of both groups is visualized in Fig. 3A. While the pupil diameters stay almost constant in the placebo group, pupil diameters decline in the THC group until 60 min and increase afterward, returning close to baseline. The statistical tests reveal a significant global time effect (*p* = 0.01), indicating that changes in pupil diameter over time were detected, whereas due to the small sample size the data do not provide evidence of a group (*p* = 0.25) ofir interaction effect between group and time (*p* = 0.29).

### Latency

The course of the latency of both groups is visualized in Fig. 3B. While latency stays almost constant in the placebo group, it increases in the THC group until 60 min. The statistical tests did however not reveal a significant global time effect (*p* = 0.49), a global group effect (*p* = 0.76), or an effect of the interaction between group and time (*p* = 0.17).

### Velocity of contraction

The course of the velocity of contraction is visualized in Fig. 3C. While the velocity of contraction stayed almost constant in the placebo group, it slightly dropped in the THC group after administration of THC and returned to baseline over time. The statistical tests did however not reveal a significant global time effect (*p* = 0.56), a global group effect (*p* = 0.80), or an effect of the interaction between group and time (*p* = 0.55).

### Constriction time

The course of the constriction time is visualized in Fig. 3D. While the constriction time remained almost constant in the placebo group, it was significantly reduced in the THC group at all timepoints after THC administration (*p* < 0.01 for all timepoints compared to baseline). There was a significant global time effect (*p* < 0.01), a significant global group effect (*p* < 0.01), and a significant global effect of the interaction between group and time (*p* < 0.01).

### Contraction amplitude

The course of the contraction amplitude is visualized in Fig. 3E. While the contraction amplitude remained almost constant in the placebo group, it was significantly reduced in the THC group at all timepoints after THC administration (*p* < 0.01 for all timepoints compared to baseline). The contraction amplitude increased between 240 and 300 min after injection, but remained reduced compared to baseline after 300 min. There was a significant global group effect (*p* < 0.01), and a trend for a global time effect (*p* = 0.06) and for a global effect of the interaction between group and time (*p* = 0.08).

**Table 3:** Pupil diameter and parameters of the pupillary light reflex. Data are mean ± sd

	Baseline	20 min	60 min	120 min	180 min	240 min	300 min
Pupil diameter (mm)*							
THC	6.09 ± 0.86	5.65 ± 0.57	5.45 ± 0.39	5.82 ± 0.57	5.86 ± 0.85	5.91 ± 0.91	5.90 ± 0.74
Placebo	6.28 ± 0.46	6.03 ± 0.59	6.11 ± 0.53	6.07 ± 0.22	6.20 ± 0.50	6.13 ± 0.44	6.20 ± 0.41
Latency (s)*							
THC	0.26 ± 0.01	0.27 ± 0.03	0.28 ± 0.03	0.28 ± 0.03	0.27 ± 0.02	0.28 ± 0.02	0.26 ± 0.02
Placebo	0.27 ± 0.01	0.26 ± 0.01	0.27 ± 0.01	0.27 ± 0.01	0.27 ± 0.02	0.27 ± 0.02	0.27 ± 0.01
Velocity of contraction (mm/s)**							
THC	4.00 ± 0.84	3.05 ± 1.55	3.39 ± 1.44	3.68 ± 1.33	3.49 ± 1.12	3.55 ± 1.08	3.91 ± 0.65
Placebo	4.72 ± 0.78	4.40 ± 0.77	4.34 ± 1.14	4.30 ± 0.85	4.54 ± 0.70	4.61 ± 0.41	4.16 ± 1.34
Constriction time (s)*							
THC	0.58 ± 0.08	0.44 ± 0.14	0.44 ± 0.14	0.44 ± 0.12	0.45 ± 0.07	0.48 ± 0.08	0.52 ± 0.09
Placebo	0.64 ± 0.16	0.65 ± 0.09	0.64 ± 0.16	0.56 ± 0.02	0.65 ± 0.19	0.61 ± 0.08	0.57 ± 0.09
Contraction amplitude (mm)**							
THC	1.38 ± 0.29	0.83 ± 0.39	0.89 ± 0.31	0.86 ± 0.30	0.94 ± 0.28	0.99 ± 0.27	1.17 ± 0.24
Placebo	1.74 ± 0.44	1.69 ± 0.32	1.55 ± 0.45	1.52 ± 0.39	1.65 ± 0.44	1.62 ± 0.32	1.45 ± 0.56

* Placebo group: *n* = 4 for all timepoints; THC group: baseline *n* = 14, 20 min *n* = 10, 60 min *n* = 7, 120 min *n* = 10, 180 min *n* = 13, 240 min *n* = 12, 300 min *n* = 13; ** Placebo group: *n* = 4 for all timepoints; THC group: baseline *n* = 14, 20 min *n* = 8, 60 min *n* = 7, 120 min *n* = 10, 180 min *n* = 13, 240 min *n* = 12, 300 min *n* = 13

## Discussion

This study assessed the pupillary reaction to light and pupil size after intravenous administration of THC in healthy volunteers not regularly consuming cannabis. It demonstrated that THC has a significant influence on the function of the pupil and dampens the relative amplitude of pupillary constriction as a key parameter of the pupillary light reflex. Pupil diameter, constriction time and contraction amplitude also demonstrated a reduction, while latency increased after THC administration. This means that the pupil’s reaction to light is slower and less pronounced in individuals under the influence of THC compared to people not under the influence of THC. The study demonstrated that the effects of THC on the eye can be detected using the simple tool of pupillography.

It has been described that other substances acting on the central nervous system can alter the pupillary light reflex and that these changes can be detected by pupillography [8–10]. It has also been described that the effects of THC might vary between regular and occasional cannabis users [6]. Here, we assessed the effect of THC on the eye in volunteers not regularly consuming cannabis. A study that was published after the start of the presented study demonstrated that smoking of cannabis in habitual cannabis consumers resulted in a weakened pupil function [16]. This is in line with the results of our study in individuals not regularly consuming cannabis. Similar to our study, some changes to parameters of the pupillary light reflex did not achieve statistical significance, but the overall effect of cannabis clearly was a dampened pupil function in the mentioned study in habitual cannabis consumers [16]. The study reported on individuals who were part of a drug substitution program and some individuals had concomitant use of other substances. The fact that both the published study assessing regular cannabis users with inhalational cannabis administration [16] and the present study including cannabis-naïve or –abstinent users with IV administration of THC demonstrate a dampened pupillary light reflex and a reduced pupil size after THC administration confirm a clear effect of THC on the pupil that is measurable using pupillography. Hence, results from the present study in cannabis-naïve or abstinent volunteers are not directly transferrable to individuals who regularly consume cannabis or other substances altering the central nervous system but are in agreement with results from these populations.

Drug testing is commonly performed in the context of fitness to drive or workplace testing. The results of such testing are often based on quantification of drugs and metabolites in blood and urine even though thresholds of measured drug concentrations are a topic of debate and there is evidence that drug concentrations in blood do not necessarily correspond to physical and mental drug effects [17, 18]. With regards to cannabis, discussions about limits of plasma levels that might not impair driving ability are ongoing. Pupillography is a simple technique which takes only seconds to complete and provides objective, reproducible measurements assessing functional drug effects. Note that while we used quantifiable methods to assess the impact of THC on pupil-related metrics and demonstrated that such effects are detectable by pupillography, we do not suggest that pupillography can predict plasma levels of THC or other physical or psychotropic effects of THC. Instead, we suggest that pupillography could be a valuable, functional addition to plasma level testing. Also, given that peak plasma concentrations occur at 1 to 5 min after THC administration [14, 19], it is evident that peak effects occur much later than peak plasma concentrations. This is in line with previous results suggesting that peak psychotropic effects occur when plasma concentrations are already declining [17, 18]. This might be linked to the distribution of THC from blood to the central nervous system and its effect sites during the rapid distribution phase [20]. The relative amplitude of pupillary constriction can be interpreted as a measure of the contractility of the pupil in response to light. This contraction is mediated by the sphincter of the pupil which is innervated by the parasympathetic nerves. Since THC primarily demonstrates parasympatholytic actions [21] the reduction in relative amplitude demonstrated by the presented data correlates well with the pharmacodynamic properties of THC. Our data demonstrate that this effect can be detected. There is, though, no defined reference range of normal values. Our data demonstrated a relative amplitude of 15.0% at 20 min after THC administration with the dose of THC chosen for the presented study. It is unclear, how these data would change with the use of higher or lower THC doses. Our data demonstrating a reduction in the relative amplitude of pupillary constriction are in line with a recent publication demonstrating a reduced pupil size variability after THC consumption [22]. 

Previous data reported inconsistent findings regarding the effect of THC on pupil size, which can at least partially be explained by heterogeneous forms of THC administration and varying degrees of bioavailability [12]. THC is often thought to cause mydriasis, which could cause impaired vision and glare. Here, we used an intravenous application of THC to have a predictable and high bioavailability and we assessed not only pupil size, but differentiated pupillographic measurements, demonstrating a slowed and decreased pupillary constriction in response to light. Another recent publication also demonstrated a reduced pupil size after THC intake, which is in line with our results [23]. Following THC consumption, vision could be impaired by glare. This is however not a result of mydriasis, but of a sluggish reaction of the pupil to light. An increased sensitivity to light as a result of THC consumption is supported by the fact that several volunteers in the THC group could not tolerate the light stimuli of the pupillographic measurements as they found them to be disturbingly bright, while this was not the case in the placebo group. This resulted in a relevant number of aborted pupillographic measurements, particularly at the early timepoints after THC administration. While some volunteers could not complete the pupillographic measurements due to very pronounced sensitivity to light, this sensitivity to light could already be seen as somewhat suggestive of THC consumption as it did not occur in the placebo group. Pronounced sensitivity to light might cause glare and might impair driving ability, but this remains speculative. Also, it is possible that the interplay of psychotropic effects and effects of THC on the eye result in the observed intolerance of bright light noted in several volunteers. Indeed, we previously reported that psychotropic and somatic effect of THC peak between 45 and 60 min after injection [19], which is in line with the highest number of impossible pupillographic measurements that occurred at 60 min. It is possible that the volunteers not tolerating the stimulus indeed exhibited the highest degree of effects of THC on the eye and that our results therefore underestimate the effects, but again, this remains speculative. Note, however, that peak effects occur later than peak plasma levels [19]. 

The pupil size before application of the light stimuli was smaller after THC administration compared to the placebo group, hence indicating that against common belief THC much rather leads to miosis and not mydriasis, similar to other drugs such as opioids, neuroleptic drugs, cholinergic drugs like pilocarpine and acetyl-cholinesterase inhibitors like neostigmine and pyridostigmine that are known to cause miosis.

We suggest that for forensic assessments the combination of an assessment of the plasma level (which demonstrates the presence of the substance) plus pupillography (with a reduced relative amplitude that demonstrates the physical drug effect) is much better suited to judge the influence of THC on driving ability or workplace considerations than one or the other alone. We believe this is particularly true since THC is a lipophilic drug and the plasma level might not always correspond to the degree of physical effects. Pupillography could therefore be used as an easy screening test for THC plus it could serve to fortify plasma testing by proving the physical effects caused by THC.

### Limitations

The very limited number of volunteers that could be included as a result of the design of the main pharmacokinetic study reduced the power to detect statistically significant effects. The study was not randomized and only single-blinded as a result of the design of the main pharmacokinetic study in which all volunteers expressing rare genetic variants of CYP2C9 received THC to allow for pharmacokinetic modelling of intravenous THC. This means that all study personnel was unblinded, but volunteers were blinded. Since effects of THC were very obvious (for both study personnel and volunteers), complete blinding would not have been possible in any way. Also, the study was not a cross-over study and hence it does not allow for within-subject comparisons. THC is usually administered orally or by inhalation, but very rarely intravenously. The intravenous route was chosen to enable pharmacokinetic/ pharmacodynamic modelling and to study the effects of THC without the confounder of the extremely variable bioavailability with the oral and the inhalational route. The first pupillographic measurement after THC administration was done after 20 min, when concentrations in arterial and venous blood can be expected to have equilibrated. This was because a lot of blood sampling was done in the first 20 min to acquire proper pharmacokinetic data and because most volunteers in the THC group were too “high” within the first 20 min to cooperate with pupillographic measurements. It is theoretically possible that with the plasma peak of THC and THC-OH pupils dilate and that this dilation had already disappeared after 20 min.

## Conclusions

This study demonstrated that THC significantly impacts the function of the pupil, reducing the relative amplitude of pupillary constriction, pupil diameter, constriction time and contraction amplitude and increasing latency. This means that THC results in a slower and less pronounced reaction of the pupil to light. These data provide evidence that pupillography, a very simple and rapidly applicable method, is able to objectively detect the effects of THC on the human eye. This can substantiate the ongoing discussions about liability following THC consumption, which is of interest to the scientific community, but also to the legislation, jurisdiction and the wider public community.

## Data Availability

The datasets used and/or analyzed during the current study are available from the corresponding author on reasonable request and with ethics committee approval and approval of the relevant authorities.
